# Clinical Significance of Additional Wide Resection for Unplanned Resection of High Grade Soft Tissue Sarcoma

**DOI:** 10.2174/1874325000802010126

**Published:** 2008-07-30

**Authors:** T Morii, H Yabe, H Morioka, U Anazawa, Y Suzuki, Y Toyama

**Affiliations:** Department of Orthopaedics Surgery, Keio University School of Medicine, 35 Shinanomachi Shinjuku, Tokyo 160-8582, Japan

## Abstract

**Purpose:**

Unplanned resection of musculoskeletal sarcoma involves tumor excision without any suspicion of malignancy or regard for the necessity of defining adequate margins. For orthopaedic oncologists, many opportunities arise for management of unplanned resections initially performed by non-specialist surgeons. The puropose of this study is to assess the clinical outcomes and the problems of the patients with unplanned resection of high-grade soft tissue sarcoma.

**Methods:**

77 consecutive patients were retrospectively reviewed. Oncological outcomes together with validity and problems of additional treatments were analyzed.

**Results:**

Five-year local recurrence-free survival, metastasis-free survival, event-free survival and total survival were 71.55%, 73.2%, 57.5% and 85.9%, respectively. Among adjuvant therapy including additional wide resection, radiotherapy and systemic chemotherapy, only additional wide resection significantly improved oncological outcomes.

**Conclusion:**

Additional wide resection appears to be effective in the treatment of high-grade soft tissue sarcomas following primary resection with compromised margins of resection.

## INTRODUCTION

Unplanned resection of soft tissue sarcoma is defined as excision without regards for the necessity to remove a margin of normal tissue covering the tumor without histological diagnosis by biopsy [1-3]. Although the principle of treatment for high-grade soft tissue sarcoma has been established as preoperative histological evaluation by biopsy and excision with adequate margin [4, 5], numerous chances still remain in specialist cancer institutes to manage soft tissue sarcoma cases following unplanned resection initially performed by non-specialist orthopaedic surgeons, general surgeons, plastic surgeons or dermatologists. The purpose of this study was to assess the following in patients with unplanned high-grade soft tissue sarcoma referred from non-specialists surgeons: 1) oncological outcomes; 2) validity of additional treatments; and 3) profile and problems of additional wide resection.

## MATERIALS AND METHODS

Subjects comprised 77 patients (39 men, 38 women; mean age, 49.3 years) who had initially undergone unplanned resection of high-grade soft tissue sarcoma by non-specialist surgeons and had been referred to our hospital because of histological diagnosis of high grade sarcoma over the past three decades. Patients with metastases at presentation were excluded, as were patients referred more than two months after initial unplanned resection. In addition, even though the surgery was performed immediately, patients with obvious palpable masses considered as macroscopic residual tumor due to incomplete resection rather than local recurrence were omitted. Finally, patients with low-grade cases were excluded from the present study due to the acceptable oncological outcomes and current controversy regarding marginal resection for this condition [6, 7].

Tumors were located in the lower extremity in 40 cases, in the upper extremity in 18 and in the trunk in 19. Mean duration of follow-up was 75 months (range, 12-240 months). Histological diagnoses were pleomorphic MFH/undifferentiated high grade pleomorphic sarcoma in 24 cases, synovial sarcoma in 18, malignant peripheral nerve sheath tumor in seven, leiomyosarcoma in seven, liposarcoma in seven and other high grade sarcoma in 14.

For 32 cases, non-surgical therapy alone was performed. Additional wide resection was performed for the remaining 45 cases. Among these, 27 cases received neither systemic chemotherapy nor radiotherapy. Chemotherapy, radiotherapy and combined chemo- and radiotherapy were performed for the remaining seven, six and five cases, respectively.

Establishment of the concept of safety margin [5] and the widespread adoption of magnetic resonance imaging (MRI) have resulted in modifications of the treatment policy for unplanned resection and the introduction of additional wide resection instead of radiotherapy in 1989 in our institute; therefore, a negative correlation between the indications for additional wide resection and radiotherapy was found in the present study. Thus, although no prospectively selected criteria were used for radiotherapy, this adjuvant therapy was indicated mainly for cases before 1989. This modality was also indicated for patients who declined additional wide resection. In addition, radiotherapy in cases of additional wide resection cases was indicated by the operating surgeons when a higher risk of recurrence was considered to exist on clinical grounds. Median total radiation dose was 40 Gy (range 20-55 Gy).

To determine margins for additional wide resection, presence of residual tumor, hemorrhage, edema or scar tissue was evaluated using magnetic resonance imaging (MRI) at presentation. If available, results of MRI for the lesion before unplanned resection were carefully inspected. T2-weighted MRI was useful in evaluation of secondary changes within muscle. Enhanced T1-weighted MRI facilitated the detection of scar tissue and residual tumor in subcutaneous tissue [8]. Hemorrhage, edema or scar tissue were thought to be contaminated with tumor and excised with adequate margin. For invasive subtypes such as undifferentiated high grade pleomorphic sarcoma or epithelioid sarcoma, curative margin was determined. For most other cases, resection with adequate wide margin was planned. Skin incision of the unplanned resection was excised with skin cuffs. Detection by residual tumor and determination of the margin in the additional wide resection specimen were performed according to the standard pathological examination as previously reported [3-5, 8].

Although recent meta-analyses have suggested some effect of chemotherapy on total survival, application of chemotherapy for high-grade soft tissue sarcoma remains controversial with the exception of round cell tumors [9-11]. If the patient accepted the proposed chemotherapy after the disclosure of controversial aspects, systemic chemotherapy was performed in the present study. Doxorubicin (60mg/m^2^/cycle) and ifosfamide (10g/m^2^/cycle) based regimens were used.

For these cases, oncological outcomes and validity of additional therapy were analyzed. Moreover, in order to extract the problems with additional wide resection, patients’ profiles, complications and validity of other adjuvant therapy combined with additional wide resection were also analyzed. Oncological outcomes were calculated using the Kaplan-Meier method [12]. The significance of differences between survival curves of populations was evaluated using log-rank testing for univariate influence and Cox stepwise regression for multivariate influence [13]. Values of p < 0.05 were considered statistically significant. The start point of the survival analysis was the date of first additional treatment. Endpoints were local recurrence, metastasis, event-free survival and total survival. Independent variables in the present study included age, sex, location, size, site, depth, pathology (round cell *vs* non-round cell), additional wide resection, radiotherapy and systemic chemotherapy.

## RESULTS

**Fig (1):**
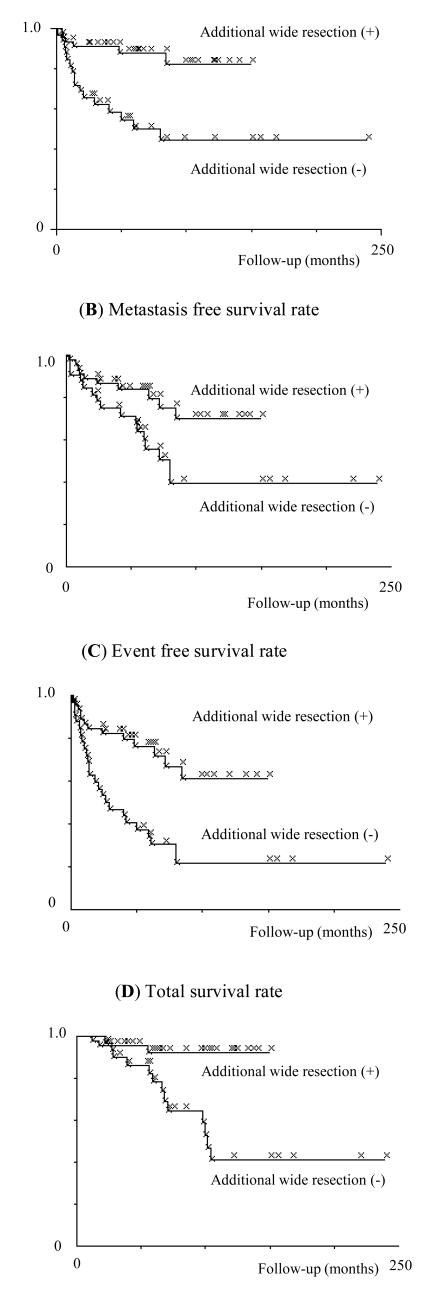
Kaplan-Meier estimates of oncological outcomes. Prognostic impact by additional wide resection on local control (**A**), metastasis free survival (**B**), event free survival (**C**) and total survival (**D**).

Five-year local recurrence-free survival, metastasis-free survival, event-free survival and total survival rates were 71.5%, 73.2%, 57.5% and 85.9%, respectively. Among the independent variables tested in this study, only application of additional wide resection was conducted to determine predictive values for local recurrence, metastasis, event-free survival and total survival (Fig. **1**, Table **1**).

In the 45 cases with additional wide resection, five-year local recurrence-free survival, metastasis-free survival, event-free survival and total survival rates were 87.9%, 83.8%, 76.3% and 92.1%, respectively. Inspection of the specimen from additional wide resection revealed the residual tumor in 22 cases (44.8%). Surgical margin was negative in 40 cases and positive in five. Plastic surgery was needed for soft tissue reconstruction after additional wide resection in 23 cases (51.1%). Combined nerve and artery resection was needed in seven cases and one case, respectively. Complications were infection in four cases, peroneal nerve palsy in two cases and compartment syndrome in one case. Regarding oncological outcomes of this subgroup, application of radiotherapy or systemic chemotherapy, residual tumor in the specimen, plastic surgery and margin of the additional wide resection did not represent significant prognostic factors. The validity of radiotherapy and chemotherapy in additional wide resection was thus not confirmed.

## DISCUSSION

The treatment of choice for cases of unplanned resection includes additional wide resection with or without radiotherapy for local control [14]. Additional wide resection has shown outstanding oncological outcomes in previous reports. For example, Zoling *et al*. reported 61 of 67 cases (91%) were alive after additional wide resection for inadequate margins [15]. Sugiura *et al*. and Masono *et al*. reported the total five-year survival rates of cases with additional wide resection as 84% and 91.3%, respectively [8, 16]. In terms of oncological outcomes, the current findings are in accordance with those previous data. The criticism has been made that the oncological outcomes for additional wide resection might include possible bias by smaller mass size, superficial location and lower grade in unplanned resection cases [8]. Moreover, patients with high-risk tumors might be referred before any treatment and will therefore undergo proper resection [17]. In the present study, sampling bias by grade was avoided by exclusion of low-grade cases. Outcomes were, however, as good as those for cases with proper conventional procedure. Although not covering samples in the trunk, Lewis *et al*. showed an interesting data that after adjusting for size, depth or grade between the definitive radical resection group and additional wide resection group followed by unplanned resection, additional wide resection represented an independent favorable variable [17]. They failed to provide clear explanations for this unexpected finding. Speculations included the broader margins from additional wide resection compared to definitive wide resection, the inhibitory effects on the micro-metastases by activating anti-angiogenic factors secreted by the primary lesion, and activation of the endogenous immune systems by residual tumor cells.

The rate of the residual tumor in additional wide resection specimens reportedly ranges from 40.4% to 57.9% [2,8,15,16]. A lack of radiological evidences of residual tumor in most specimens from additional wide resection has been emphasized [16]. These issues support the immediate application of additional wide resection to cases of unplanned resection. The impact of residual tumor on oncological outcomes remains controversial. Residual tumor has been reported as a risk for local recurrence [2] and disease free survival [8]. Conversely, data from Masono [16] and the current study failed to show that the residual tumor was an independent risk factor for local recurrence or total survival. Candidate biases for this discrepancy include: differences among the institutions in the quality and methods of the histological examination; differences in the independent variables such as histology or grade; differences in the adjuvant therapies; and differences in the quality of the additional wide resection procedure.

Additional wide resection requires a broader margin than in conventional wide resection. This may lead to the need for soft tissue reconstruction, prolonged operation time, complications and functional loss. Rates of wound complication as high as 24% and rates of re-operation chance as high as 18% have been reported [16]. Difficulties in determining margins after unplanned resection have also been noted [8]. The present study showed considerable high rate of combined nerve resection. All these issues suggested the need for reduction in the chance of additional wide resection for unplanned resection cases by application of proper conventional wide resection as initial treatment for high-grade soft tissue sarcomas. Thus management principles of soft tissue tumor must be widespread among the whole physicians providing primary treatments for the disease.

In the current study, cases with radiotherapy showed a tendency toward worse oncological outcomes. As the treatment policy was changed in 1989 in our institute, as mentioned above, the negative correlation between indications for additional wide resection and for radiotherapy was marked. Moreover, the dose in the present study was under 50Gy most cases. Radiation dose for successful control of the soft tissue sarcoma with positive margins or unplanned resection is reported as over 65 Gy [14,18,19]. We thus acknowledge bias in this retrospective study as to the efficacy of radiotherapy.

We failed to confirm the functional outcome of unplanned resection cases due to the lack of the data in early cases. Although functional loss following additional wide resection might be plausible, no significant evidence has been reported. Mean total function using Enneking’s system [20] has been reported as ranged from 83.3% to 94.7% [8, 16], suggesting acceptable function. Despite a trend toward a poorer functional outcome in the presence of infection, there was no significant factor that leads poorer function [16]. These data supported the application of additional wide resection in terms of functional results.

## Figures and Tables

**Table 1.** 

Endpoints	Significant Predictable Variables in Univariate Analysis	Survival Number	Five Year Survival Rate	Univariate Analysis	Multivariate Analysis
				p Value	Risk Ratio (exp) Range p Value
Local recurrence	Additional wide resection (+) (-)	39/45 16/32	87.90% 49.9%	0.0007	4.4 1.7-11.2 0.002
Metastasis	Additional wide resection (+) (-)	35/45 16/32	83.80% 60.00%	0.03	2.4 1.1-5.3 0.03
Events	Additional wide resection (+) (-)	32/45 8/32	76.30% 34.10%	0.0003	3.2 1.6-6.3 0.0007
Total survival	Additional wide resection (+) (-)	42/45 19/32	92.10% 78.30%	0.0002	4.7 1.2-17.8 0.02
	Radiotherapy (+) (-)	22/33 39/44	75.60% 94.10%	0.04	0.62 0.2-1.9 0.41

## References

[1] Giuliano AE, Eilber FR (1985). The rationale for planned reoperation after unplanned total excision of soft-tissue sarcomas. J Clin Oncol.

[2] Davis AM, Kandel RA, Wunder JS (1997). The impact of residual disease on local recurrence in patients treated by initial unplanned resection for soft tissue sarcoma of the extremity. J Surg Oncol.

[3] Noria S, Davis A, Kandel R (1996). Residual disease following unplanned excision of soft-tissue sarcoma of an extremity. J Bone Joint Surg Am.

[4] Kawaguchi N, Ahmed AR, Matsumoto S, Manabe J, Matsushita Y (2004). The concept of curative margin in surgery for bone and soft tissue sarcoma. Clin Orthop Relat Res.

[5] Kawaguchi N, Matumoto S, Manabe J (1995). New method of evaluating the surgical margin and safety margin for musculoskeletal sarcoma, analysed on the basis of 457 surgical cases. J Cancer Res Clin Oncol.

[6] Kubo T, Sugita T, Shimose S, Arihiro K, Ochi M (2006). Conservative surgery for well-differentiated liposarcomas of the extremities adjacent to major neurovascular structures. Surg Oncol.

[7] Gerrand CH, Wunder JS, Kandel RA (2001). Classification of positive margins after resection of soft-tissue sarcoma of the limb predicts the risk of local recurrence. J Bone Joint Surg Br.

[8] Sugiura H, Takahashi M, Katagiri H (2002). Additional wide resection of malignant soft tissue tumors. Clin Orthop Relat Res.

[9] Pervaiz N, Colterjohn N, Farrokhyar F, Tozer R, Figueredo A, Ghert M (2008). A systematic meta-analysis of randomized controlled trials of adjuvant chemotherapy for localized resectable soft-tissue sarcoma. Cancer.

[10] Karavasilis V, Seddon BM, Ashley S, Al-Muderis O, Fisher C, Judson I (2008). Significant clinical benefit of first-line palliative chemotherapy in advanced soft-tissue sarcoma: retrospective analysis and identification of prognostic factors in 488 patients. Cancer.

[11] Grier HE, Krailo MD, Tarbell NJ (2003). Addition of ifosfamide and etoposide to standard chemotherapy for Ewing's sarcoma and primitive neuroectodermal tumor of bone. N Engl J Med.

[12] Kaplan EL, Meier P (1958). Nonparametric estimation from incomplete observations. J Am Statist Assoc.

[13] Cox DR (1972). Regression models and life tables. J R Stat Soc Series B.

[14] Kepka L, Suit HD, Goldberg SI (2005). Results of radiation therapy performed after unplanned surgery (without re-excision) for soft tissue sarcomas. J Surg Oncol.

[15] Zornig C, Peiper M, Schroder S (1995). Re-excision of soft tissue sarcoma after inadequate initial operation. Br J Surg.

[16] Manoso MW, Frassica DA, Deune EG, Frassica FJ (2005). Outcomes of re-excision after unplanned excisions of soft-tissue sarcomas. J Surg Oncol.

[17] Lewis JJ, Leung D, Espat J, Woodruff JM, Brennan MF (2000). Effect of reresection in extremity soft tissue sarcoma. Ann Surg.

[18] Zagars GK, Ballo MT (2003). Sequencing radiotherapy for soft tissue sarcoma when re-resection is planned. Int J Radiat Oncol Biol Phys.

[19] Delaney TF, Kepka L, Goldberg SI (2007). Radiation therapy for control of soft-tissue sarcomas resected with positive margins. Int J Radiat Oncol Biol Phys.

[20] Enneking WF, Dunham W, Gebhardt MC, Malawar M, Pritchard DJ (1993). A system for the functional evaluation of reconstructive procedures after surgical treatment of tumors of the musculoskeletal system. Clin Orthop Relat Res.

